# The Treatment of Epilepsy

**Published:** 1906-02-17

**Authors:** 


### THE TREATMENT OF EPILEPSY.

Dr. Edwin Bramwell has written a useful contribution to this subject. The first step after the diagnosis has been made is to conduct an exact inquiry into the patient's general health, and to remedy any defect in this direction. Equally important is the endeavour to detect any circumstance which appears to act as the exciting cause of the fits, such, for example, as indigestion, constipation, excitement, etc. Another claim is an attempt to detect any peripheral source of irritation and to remove this if possible. Adenoids, an adherent prepuce, an aural or nasal polypus, carious teeth, errors of refraction, and intestinal worms may be mentioned in this connection. Though occasionally these conditions may act as exciting causes, it is probable that the range of their influence has been exaggerated. An important element in the active treatment of epilepsy is precise regulation of the patient's habits, so that order, regularity, and moderation shall be ruling factors in his life. The most suitable diet for epileptics has given rise to much discussion, but there is no valid reason for restriction beyond the limitation of red meat to one meal per diem, and the avoidance of over-eating and of foods known by experience to disagree with the patient. Even the direction to omit sodium chloride from the dietary, and to use bromides in its place, is of doubtful value, and has probably no superiority over the administration of bromides in the usual fashion. Alcohol should be forbidden. Out-door exercise is to be encouraged, though forms of it, where the occurrence of a fit would mean grave danger—for example, swimming, boating, etc.—can only be permitted under adequate supervision. The education difficulty in the case of epileptic children is a considerable one. With anything like frequent and severe fits, school is out of the question. All the same it is in the patient's interest that his mind should be kept regularly occupied, and that he should be properly educated, and it would be well were special schools or classes formed for these unfortunate children. Regarding the question of marriage, it may be allowed that matrimony will probably produce little effect in the number or severity of the fits, but the danger of epilepsy, imbecility, or insanity in one or more of the offspring is considerable.

Drug treatment requires a careful study of the individual case and the selection of a line of treatment, both qualitative and quantitative, which will produce the best effect with a minimum dosage. This can hardly be determined except by one or more therapeutic experiments, but whatever selection be made it should be given a thorough and methodical trial, and an exact note should be made of the occurrence of the fits as a guide to an estimate of the effects of the remedy. The bromides are the agents on which most reliance is placed, the potassium salt being that most generally used; some prefer the bromide of sodium or ammonium, whilst others combine the three salts. The usual dose is 10 to 15 grains twice a day, twenty minutes after food, with double the amount at bedtime. When there are merely nocturnal attacks the evening dose may be sufficient, whilst a tendency to repetition of the attack soon after rising should be met by a dose of bromide as soon as the patient wakes. In individual instances the above doses must be considerably increased, but there is little or no advantage in exceeding a maximum of 90 grains in the 24 hours. If, under the dose selected, the fits cease, the prescription must be steadily maintained; should they be merely diminished a corresponding dose of sodium, ammonium, or strontium bromide should be substituted in the hope of further improvement; whilst failure calls for the addition of other remedies. Among these borax occupies a prominent place, more especially perhaps where petit mal is a conspicuous feature of the case. It may be given alone or together with the bromides in doses of 5 to 10 grains, and gradually increased. It sometimes causes gastro-intestinal symptoms, and is said occasionally to produce psoriasis. Digitalis is another remedy that may be added when the bromides prove unsuccessful, and the same is true of tincture of belladonna (5 to 10 mins.) and liquor trinitrini (1 to 2 mins.). Zinc-oxide in pill form ($2\frac{1}{2}$ to 5 grains or more) two or three times a day is often useful, particularly in the treatment of petit mal. Potassium iodide is of great service where there is a syphilitic history, and is often useful in the epilepsies of later life where there is arterio-sclerosis, and where bromides rarely prove successful. Perseverance and regularity of administration are essential, and this rule must be enforced for at least two years after the cessation of the fits, though during the second year the dose of the remedies may be greatly reduced.

